# The use of adjuvant chemotherapy is not associated with recurrence or cancer-specific death following curative resection for stage III rectal cancer: a competing risks analysis

**DOI:** 10.1186/s12957-023-03021-w

**Published:** 2023-05-18

**Authors:** Kheng-Seong Ng, Charles Chan, Matthew John Francis Xavier Rickard, Anil Keshava, Peter Stewart, Pierre Henri Chapuis

**Affiliations:** 1 Colorectal Surgical Unit, Concord Repatriation General Hospital, Sydney, NSW 2139 Australia; 2 Sydney Medical School, Concord Institute of Academic Surgery, The University of Sydney, Sydney, NSW 2006 Australia; 3 Division of Anatomical Pathology, Concord Repatriation General Hospital, Sydney, NSW 2139 Australia; 4 Concord Clinical School, Sydney Medical School, The University of Sydney, Sydney, NSW 2006 Australia

**Keywords:** Rectal cancer, Adjuvant chemotherapy, Disease recurrence, Cancer-specific death, Competing risks analysis

## Abstract

**Background:**

The role of adjuvant chemotherapy (AC) in stage III rectal cancer (RC) has been argued based on evidence from its use in colon cancer. Previous trials have analysed disease-free and overall survivals as endpoints, rather than disease recurrence. This study compares the competing risks incidences of recurrence and cancer-specific death between patients who did and did not receive AC for stage III RC.

**Methods:**

Consecutive patients who underwent a potentially curative resection for stage III RC (1995–2019) at Concord Hospital, Sydney, Australia, were studied. AC was considered following multidisciplinary discussion. Primary outcome measures were the competing risks incidences of disease recurrence and cancer-specific death. Associations between these outcomes and use of AC (and other variables) were tested by regression modelling.

**Results:**

Some 338 patients (213 male, mean age 64.4 years [SD12.7]) were included. Of these, 208 received AC. The use of AC was associated with resection year (adjusted OR [aOR] 1.74, 95%CI 1.27–2.38); age ≥75 years (aOR0.04, 95%CI 0.02–0.12); peripheral vascular disease (aOR0.08, 95%CI 0.01–0.74); and postoperative abdomino-pelvic abscess (aOR0.23, 95%CI 0.07–0.81). One hundred fifty-seven patients (46.5%) were diagnosed with recurrence; death due to RC occurred in 119 (35.2%). After adjustment for the competing risk of non-cancer death, neither recurrence nor RC-specific death was associated with AC (HR0.97, 95%CI 0.70–1.33 and HR0.72, 95%CI 0.50–1.03, respectively).

**Conclusion:**

This study found no significant difference in either recurrence or cancer-specific death between patients who did and did not receive AC following curative resection for stage III RC.

## Background

The use of adjuvant chemotherapy (AC) following resection of stage III rectal cancer (RC) has been recommended based largely on randomised trial evidence of favourable outcomes from its use in colon cancer [1]. Nonetheless, the use of AC in stage III RC remains controversial [2, 3]. While a Cochrane meta-analysis demonstrated a significant benefit for AC both in terms of disease-free survival (DFS) and overall survival (OS) [4], a subsequent systematic review comparing AC versus observation following surgery alone in locally advanced RC [5] reported improved OS in the chemotherapy group in only two of six studies [6–12]. Notably, both of these reviews were limited by their inability to analyse outcomes by tumour stage (i.e. stage II versus stage III tumours). Moreover, many of the included trials were conducted prior to total mesorectal excision (TME) being implemented in routine care, whilst only in a few trials was multimodal treatment, including neoadjuvant (chemo)radiotherapy, actually delivered. Based on this, it has been argued that the results from these systematic reviews based mainly from retrospective evidence [13] cannot apply to contemporary RC management as they include patients with a substantially higher risk of both local pelvic and systemic recurrence, thus potentially enhancing the likelihood of AC impacting favourably on survival [14].

Recently, the efficacy of AC in protecting against recurrence in stage III colon cancer has been questioned [15]. Suggested reasons why such treatment may no longer be beneficial include increasing adoption of a standardized technique for resecting colon cancer, improvements in the assessment of the extent of tumour burden present prior to surgery using modern imaging methods, and the introduction of high-quality multidisciplinary patient care. In routine clinical practice at Concord Hospital, we have previously shown no significant difference in the competing risks incidence of either recurrence or colon-cancer-specific death between stage III patients who received AC and those who did not [16]. Similarly, it is conceivable that the efficacy of AC in stage III RC may be also influenced by the same factors previously noted, particularly by the increasing adoption of TME [17], as none of the previously mentioned randomised trials in RC indicate the surgical technique employed [6–12].

Previous studies on the topic have also been limited by their assessment of disease recurrence using so-called naïve Kaplan–Meier censoring. If recurrence and cancer-specific death are assessed by Kaplan–Meier analyses [18] in which patients who die of causes other than RC have their survival times censored, this violates the assumption that censored patients would continue to have the same risk of recurrence and cancer-specific death as if they had not died of the other cause [18–20]. Obviously, patients who die of another cause cannot continue to be at risk of recurrence or death due to RC. The consequence of this violation is incorrect estimates of the incidence of recurrence and RC-specific death rates. Competing risks techniques will produce more accurate and precise estimates of recurrence and cancer-specific death rates and should be the modelling approach for such survival analyses.

The aim of this study was to compare the competing risks incidence of recurrence and RC-specific death between patients who did and did not receive AC and therefore clarify any additional (and potentially causal) beneficial association with the use of AC on long-term outcomes following resection. Our hypothesis was that oncological outcomes would be significantly better in those who received chemotherapy than in those who did not.

## Methods

This study was based on analysis of a prospective database of consecutive patients with RC who were operated on by specialist colorectal surgeons, all members of the Concord Hospital Colorectal Surgical Unit [21, 22]. The data set included patient characteristics, comorbidity, mode of presentation, imaging, surgical management, postoperative complications, pathology and follow-up and has the approval of the Sydney Local Health District Ethics Committee (CH62/62011-136-P Chapuis HREC/11/CRGH206).

All resections were performed by mobilisation of the rectum along strict anatomical planes employing a standardized technique adopted in 1981 [23], equivalent to the technique of TME of the rectum as promoted by Heald et al. [17]. Resections performed between 1995 and 2019 inclusive were selected for analysis. Comorbidity and complications were as defined previously [24]. Examination of the resected specimen was performed or reviewed by one pathologist (CC) with a special interest in colorectal cancer using a standard protocol [25]. Only adenocarcinomas (including mucinous and signet ring carcinomas) of the rectum (defined as those tumours whose distal edge was within 15 cm from the anal verge) were included in the data set. All pathological characteristics analysed were looked for in every specimen and their presence or absence was recorded explicitly. Analysis was confined to rectal tumours with nodal metastasis, no identifiable systemic disease (pTNM stage III) and no histological confirmation of tumour transection present in the proximal, distal or deep lines of resection in the operative specimen. Such patients were defined as having had a potentially curative resection. There were no missing data on any variable except cause of death (five patients), cardiac morbidity (21 patients because scoring on the New York Heart Association classification requires that the patient be mobile and not demented) and an American Society of Anesthesiologists (ASA) score (one patient not recorded). The modal category was substituted in the missing ASA score variable, whilst missing cardiac morbidity data were excluded in relevant analyses; these had no material effect on the results. Patients were excluded if they had had a prior colorectal cancer resected, or if they had inflammatory bowel disease, familial adenomatous polyposis coli or if their cause of death was unknown.

Patients without distant metastasis but with clinically suspected locally advanced RC (typically with a threatened or involved circumferential resection margin, anterior tumour position or mesorectal or side wall nodal involvement) were identified by either computed tomography, magnetic resonance imaging or endorectal ultrasonography and were considered for preoperative radiotherapy with or without supplementary chemotherapy.

AC was considered routinely for all patients at a multidisciplinary team meeting, but prescribed selectively taking into account the patient’s age, comorbidity, the presence of adverse pathological features [26], postoperative complications, social circumstances and the patient’s wishes. The chemotherapy regimens utilised varied but were in accordance with best practice at the time and for the most part were bolus injections of 5-fluorouracil and folinic acid administered daily in 5-day blocks and repeated every month for six months (Mayo Clinic regimen [27]); 5-FU and leucovorin repeated weekly for six doses with a 2-week rest between (Roswell Park regimen [28]); semi-monthly 22-h 5-FU infusion with leucovorin (de Gramont [29]); or modified oxaliplatin, folinic acid and 5-FU every 2 weeks (FOLFOX [30]). As the database was originally oriented principally towards surgical treatment, no record was kept of reason/s why individual patients did or did not receive chemotherapy or of the chemotherapy agents or regimens used in individual patients or of dose alteration, complications, interruption or cessation of treatment.

### Follow-up and assessment of recurrence and mortality

The primary outcome variables were the competing risks incidence of recurrence (local-only, systemic-only, or local plus systemic) and frequency of rectal-cancer-specific death (RC death) with death due to causes other than RC (non-RC death) adjusted as a risk competing with these outcomes.

Patients were reviewed at six monthly visits for the first 2 years after resection and yearly thereafter until death or December 31, 2020. Surveillance included clinical examination, chest X-ray, CT scan of the thorax/abdomen/pelvis, liver function tests, CEA and either sigmoidoscopy or colonoscopy for those who had had a restorative operation. Recurrence was defined as clinically or radiologically suspected (biopsy proven whenever possible) tumour in the peritoneal cavity or newly diagnosed distant metastasis. The occurrence, date and cause of death were ascertained by one of us (PC) principally from hospital records, in consultation with the patient’s surgeon or family physician, or the national death registration system. The underlying cause of death was coded according to the International Statistical Classification of Diseases and Related Health Problems—Tenth Revision.

### Statistical analysis

The statistical significance of differences between percentages was evaluated by the exact chi-squared test and difference between means were evaluated by the *F* test. Logistic regression was used in multivariable modelling of variables thought to be potentially relevant to the administration of AC. Analyses of recurrence and RC-specific death were by the competing risks method of Fine and Gray [31] as implemented in the Stata statistical package, rather than the naïve Kaplan–Meier [18]. Both OS and DFS were calculated by the Kaplan–Meier (product limit) method. The date of resection was the starting point for follow-up times. In competing risks analyses, the terminal events were recurrence at any site or death due to RC. Times were censored at last contact for patients who did not experience the terminal event and were either lost to follow-up or remained alive. To simplify interpretation of odds ratios and hazard ratios, all covariates were dichotomised at conventional cutting points or at/near the median. Covariates with a bivariate association significant at *P* ≤ 0.2 were entered into multivariable regression models. The level for two-tailed statistical significance was *P* ≤ 0.05 with confidence intervals (CI) at the 95% level. Analyses were conducted with Stata version 15 (Stata Corp, College Station, TX, 2015).

## Results

From 1203 RC resections performed in the period 1995 to 2019 inclusive, successive exclusions were 835 tumours considered not stage III, six patients with previous colorectal cancer, seven who had developed a new primary, six with a synchronous cancer, six with inflammatory bowel disease or familial adenomatous polyposis coli and five patients whose cause of death was unknown. There were 338 patients remaining with a single RC suitable for analysis. The clinical characteristics of patients who had AC are compared with those who did not in Table 1. By the close of study in December 2020, 178 patients (52.7%) had died; the median time to death was 3.3 years (range 0.01–20.9). Of the 160 patients remaining alive or lost to follow-up, the median survival time was 11.1 years (range 0.4–22.5).

**Table 1:** Clinical and pathological characteristics of patients studied

Characteristic	No adjuvant chemotherapy Number (%) or mean [sd] *n* = 130	Adjuvant chemotherapy Number (%) or mean [sd] *n* = 208	Chi-squared *P* or *F* test *P*
Male sex	78 (60.0)	135 (64.9)	0.364
Mean age (years)	71.8 [12.7]	59.8 [10.4]	*<0.001*
Age ≥ 75 years	62 (47.7)	9 (4.3)	*<0.001*
Preoperative radiotherapy	20 (15.4)	60 (28.9)	*0.005*
Resection at urgent operation	2 (1.5)	3 (1.4)	0.943
ASA physical status:			
I healthy patient	22 (16.9)	60 (28.9)	*<0.001*
II mild systemic disease	67 (51.5)	125 (60.1)	
III/IV severe systemic disease, severe systemic disease, a threat to life	41 (31.5)	23 (11.1)	
Tumour site:			
1 to 5 cm from anal verge	31 (23.9)	47 (22.6)	0.329
6 to 10 cm	68 (52.3)	96 (46.2)	
11 to 15 cm	31 (23.9)	65 (31.3)	
Surgical access:			
Open	109 (83.9)	149 (71.6)	*0.010*
Laparoscopic or laparoscopic-assisted	21 (16.2)	59 (28.4)	
Operation type			
Sphincter-preserving (anterior resection, Hartmann’s resection)	101 (77.7)	164 (78.9)	0.802
Non-sphincter preserving (abdominoperineal excision of rectum [APER])	29 (22.3)	44 (21.2)	
Mean tumour maximum surface dimension (cm)	4.5 [1.8]	4.0 [1.6]	*0.010*
Tumour maximum surface dimension ≥ 4.5 cm	66 (50.8)	80 (38.8)	*0.032*
Histological type:			
Adenocarcinoma	120 (92.3)	193 (92.8)	0.288
Mucinous	7 (5.4)	14 (6.7)	
Signet ring	3 (2.3)	1 (0.5)	
Direct spread			
Submucosa	8 (6.2)	9 (4.3)	0.595
Muscularis propria	19 (14.6)	40 (19.2)	
Beyond muscularis propria	89 (68.5)	141 (67.8)	
To serosa	14 (10.8)	18 (8.7)	
Mean number of lymph nodes examined	16 [7.9]	18 [7.8]	*0.037*
≥ 12 nodes examined	96 (73.9)	167 (80.3)	0.166
≥ 20% of nodes involved tumour	49 (37.7)	90 (43.3)	0.311
Lymph node metastasis			
Local only	125 (96.2)	196 (94.2)	0.431
Local and apical	5 (3.9)	12 (5.8)	
Apical only	0	0	
Differentiation			
Well/moderate	100 (76.9)	163 (78.4)	0.756
Poor	30 (23.1)	45 (21.6)	
Lymphovascular invasion			
Absent	73 (56.2)	117 (56.3)	0.986
Present	57 (43.9)	91 (43.8)	
Perineural invasion			
Absent	101 (77.7)	145 (69.7)	0.109
Present	29 (22.3)	63 (30.3)	
Tumour perforation			
Absent	129 (99.2)	199 (95.7)	0.06
Present	1 (0.8)	9 (4.3)	
Tumour position			
Anterior (including circumferential)	69 (53.1)	94 (45.2)	0.158
Other	61 (46.9)	114 (54.8)	
Adjacent structures taken en bloc			
No	114 (87.7)	188 (90.4)	0.435
Yes	16 (12.3)	20 (9.6)	

Italicized *P* values indicate *P* significance at ≤0.05

### Features associated with administration of adjuvant chemotherapy

Of the 338 patients, 208 (61.5%) received AC and 130 (38.5%) did not. Because reasons for not administering chemotherapy to individual patients were not initially recorded in the database, we examined the associations between chemotherapy and 37 other variables concerning background characteristics, comorbidity, perioperative conditions and events, postoperative complications and pathology, all of which we considered to be potential reasons for non-administration. Data were available on 13 other possibly relevant variables but these were not included because, in every case, the frequency of occurrence of the outcome thought likely to be associated with non-administration of chemotherapy was less than 10 (3.0%) and therefore unlikely to be clinically important.

Although several variables had a significant bivariate association with administration of chemotherapy (Table 2), logistic regression showed that only four had independent effects after adjustment for other covariates. Use of chemotherapy varied over time but its use significantly increased over the study period (Table 2). Patients aged 75 years and older at the time of surgery were less likely to receive chemotherapy as were patients with peripheral vascular disease or those who had developed a postoperative intra-abdominal or pelvic abscess/haematoma (Table 2). Notably, none of the 10 pathology characteristics was related to administration of AC.

**Table 2:** Patients having adjuvant chemotherapy (AC) by background variables, comorbidity, perioperative variables, postoperative complications and pathology

	Category^a^	Number (%) having AC	Chi^2^ *P*	Bivariate OR (95% CI)	Multivariable OR (95% CI)	Wald *P*
Background variables						
Year of resection	1995–1999	35/79 (44.3)	*0.001*	1.42	1.74 (1.27–	*0.001*
	2000–2004	48/83 (57.8)		(1.19–1.69)	2.38)	
	2005–2009	57/83 (68.7)				
	2010–2014	32/45 (71.1)				
	2015–2019	36/48 (75.0)				
Sex	Female	73/125 (58.4)	0.364	REF	–	–
	Male	135/213 (63.4)		1.23 (0.78–1.94)		
Age	< 75 years	199/267 (74.5)	*<0.001*	REF		*<0.001*
	≥ 75 years	9/71 (12.7)		0.05 (0.02–0.11)	0.04 (0.02–0.12)	
Comorbidity						
Cardiac disease	No	179/249 (71.9)	*<0.001*	REF		0.280
	Yes	26/68 (38.2)		0.24 (0.14–0.42)	0.62 (0.26–1.47)	
Respiratory disease	No	172/277 (62.1)	0.655	REF	–	–
	Yes	36/61 (59.0)		0.88 (0.50–1.55)		
Renal disease	No	197/312 (63.1)	*0.036*	REF		0.346
	Yes	11/26 (42.3)		0.43 (0.19–0.96)	0.54 (0.15–1.96)	
Diabetes (type I or type II)	No	182/293 (62.1)	0.578	REF	–	–
	Yes	26/45 (57.8)		0.83 (0.44–1.58)		
Cerebrovascular accident	No	203/320 (63.4)	*0.002*	REF		0.201
	Yes	5/18 (27.8)		0.22 (0.08–0.64)	0.39 (0.09–1.65)	
Peripheral vascular disease	No	207/322 (64.3)	*<0.001*	REF		*0.026*
	Yes	1/16 (6.3)		0.04 (0.005–0.28)	0.08 (0.01–0.74)	
Hypertension	No	137/202 (67.8)	*0.004*	REF		0.396
	Yes	71/136 (52.2)		0.52 (0.33–0.81)	1.36 (0.67–2.74)	
Perioperative variables						
Preoperative radiotherapy	No	148/258 (57.4)	*0.005*	REF		0.851
	Yes	60/80 (75.0)		2.23 (1.27–3.92)	0.92 (0.41–2.10)	
ASA	I	60/82 (73.2)	*<0.001*	0.45	0.82 (0.48–	0.487
	II	125/192 (65.1)		(0.32–	1.42)	
	III–IV	23/64 (35.9)		0.65)		
Tumour site	1–5 cm	47/78 (60.3)	0.329	0.84	–	–
	6–10 cm	96/164 (58.5)		(0.62–		
	11–15 cm	65/96 (67.7)		1.15)		
Surgical access	Open	149/258 (57.8)	*0.010*	REF		0.294
	Laparoscopic	59/80 (73.8)		2.06 (1.18–3.58)	1.75 (0.62–4.96)	
Perioperative transfusion	No	182/274 (66.4)	*<0.001*	REF		0.480
	Yes	26/64 (40.6)		0.35 (0.20–0.60)	0.74 (0.32–1.70)	
Operation type	Sphincter preserving	164/265 (61.9)	0.802	REF	–	–
	Non-sphincter preserving	44/73 (60.3)		0.93 (0.55–1.59)		
Other organ taken en bloc	No	188/302 (62.3)	0.435	REF	–	–
	Yes	20/36 (55.6)		0.76 (0.38–1.52)		
Blood loss	< 500 cc	193/309 (62.5)	0.256	REF	–	–
	≥ 500 cc	15/29 (51.7)		0.65 (0.30–1.38)		
Duration of operation	< 4 h	81/144 (56.3)	0.085	REF	–	
	≥ 4 h	127/194 (65.5)		1.47 (0.95–2.30)		
Postoperative complications						
Wound complication	No	197/314 (62.7)	0.101	REF	–	–
	Yes	11/24 (45.8)		0.50 (0.22–1.16)		
Intra-abdominal or pelvic abscess/haematoma	No	198/311 (63.7)	*0.006*	REF		*0.022*
	Yes	10/27 (37.0)		0.34 (0.15–0.76)	0.23 (0.07–0.81)	
Urinary complication	No	192/298 (64.4)	*0.003*	REF		0.282
	Yes	16/40 (40.0)		0.37 (0.19–0.72)	0.59 (0.22–1.55)	
Anastomotic leak	No	153/235 (65.1)	0.099	REF	–	–
	Yes	5/12 (41.7)		0.38 (0.12–1.24)		
Prolonged postoperative ileus	No	183/290 (63.1)	0.146	REF	–	–
	Yes	25/48 (52.1)		0.64 (0.34–1.17)		
Respiratory complication requiring consultation	No	189/297 (63.6)	*0.033*	REF		0.713
	Yes	19/41 (46.3)		0.49 (0.26–0.95)	1.22 (0.42–3.54)	
Cardiac complication	No	192/293 (65.5)	*<0.001*	REF		0.808
	Yes	16/45 (35.6)		0.29 (0.15–0.56)	1.14 (0.39–3.39)	
Early reoperation	No	200/314 (63.7)	*0.003*	REF		0.533
	Yes	8/24 (33.3)		0.29 (0.12–0.69)	0.65 (0.17–2.53)	
Pathology						
Tumour greatest luminal dimension	< 4.5 cm	126/190 (66.3)	*0.032*	REF		0.557
	>/= 4.5 cm	80/146 (54.8)		0.62 (0.40–0.96)	0.83 (0.44–1.55)	
Pathological type	Adenocarcinoma	193/313 (61.7)	0.869	REF	–	–
	Mucinous/signet ring	15/25 (60.0)		0.93 (0.41–2.14)		
T4 tumour	No	188/299 (62.9)	0.162	REF	–	–
	Yes	20/39 (51.3)		0.62 (0.32–1.21)		
Tumour involving apical node	No	196/321 (61.1)	0.431	REF	–	–
	Yes	12/17 (70.6)		1.24 (0.73–2.11)		
Percent of nodes involved	< 20%	118/199 (59.3)	0.311	REF	–	–
	≥ 20%	90/139 (64.8)		1.26 (0.81–1.97)		
Differentiation	Well/moderate	163/263 (62.0)	0.756	REF	–	–
	Poor	45/75 (60.0)		0.92 (0.54–1.56)		
Lymphovascular invasion	No	117/190 (61.6)	0.986	REF	–	–
	Yes	91/148 (61.5)		0.99 (0.64–1.55)		
Perineural invasion	No	145/246 (58.9)	0.109	REF		
	Yes	63/92 (68.5)		1.51 (0.91–2.51)		
Tumour perforation	No	199/328 (60.7)	0.060	REF		
	Yes	9/10 (90.0)		5.83 (0.73–46.60)		
Anterior tumour position	No	114/175 (65.1)	0.158	REF		
	Yes	94/163 (57.7)		0.73 (0.47–1.13)		

Italicized *P* values indicate *P* significance at ≤0.05

*OR* odds ratio, *CI* confidence interval

^a^In each cell, first line is the reference category coded 0, second line is the category of interest coded 1

### Recurrence

There were 157 patients (46.5%) who had a recurrence at any site, 50 (14.8%) who died of non-cancer causes and 131 (38.8%) who remained alive without recurrence at the close of study (Table 3). For the 208 patients who had chemotherapy, these numbers were 94 (45.2%), 13 (6.3%) and 101 (48.6%), respectively, and for the 130 who did not, 63 (48.5%), 37 (28.5%) and 30 (23.1%).

**Table 3:** Association between adjuvant chemotherapy (AC) and recurrence of rectal cancer and between other variables independently associated with administration of AC and recurrence, after adjustment for the competing risk of death due to causes other than rectal cancer

	Category^a^	Censored *n* = 131	Died of non-RC *n* = 50	Recurred *n* = 157	Bivariate hazard ratio (95% CI)	Wald *P*
Adjuvant chemotherapy	No	30	37	63	REF	0.830
	Yes	101	13	94	0.97 (0.70–1.33)	
Year of resection	1995–1999	17	14	48	0.92 (0.80–1.05)	0.206
	2000–2004	29	20	34		
	2005–2009	34	10	39		
	2010–2014	20	4	21		
	2015–2019	31	2	15		
Age	< 75 years	117	24	126	REF	0.501
	≥ 75 years	14	26	31	0.87 (0.59–1.30)	
Peripheral vascular disease	No	130	45	147	REF	0.259
	Yes	1	5	10	1.39 (0.78–2.48)	
Intra-abdominal or pelvic abscess/haematoma	No	119	45	147	REF	0.336
	Yes	12	5	10	0.73 (0.39–1.38)	

Italicized *P* values indicate *P* significance at ≤ 0.05

*RC* rectal cancer, *CI* confidence interval

^a^In each cell, first line is the reference category coded 0, second line is the category of interest coded 1

**Fig. 1:**
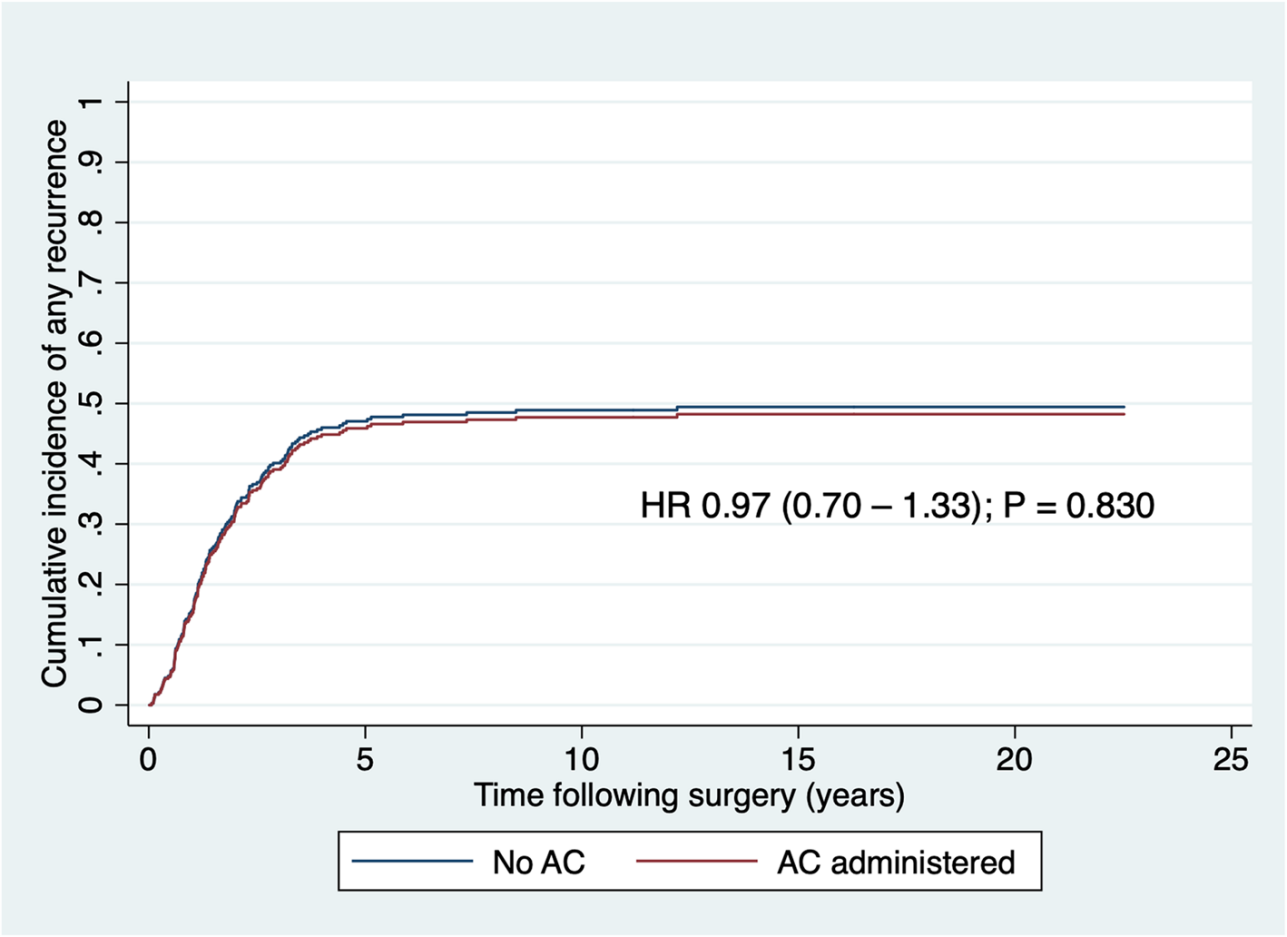
Cumulative incidence of any recurrence after potentially curative resection of rectal cancer by adjuvant chemotherapy

After adjustment for the competing risk of non-cancer death, recurrence was not significantly associated with AC, as shown in Table 3 and Fig. 1. As it was possible that one or more of the variables found to be independently associated with receiving chemotherapy could exert a suppressor effect on the association between chemotherapy and recurrence, we considered a competing risks regression model which specifically incorporated these variables and AC as predictors of recurrence. It was found that none of these variables was significantly associated with recurrence (Table 3) and therefore none could exert a suppressor effect. To demonstrate this, a multivariable regression model was created incorporating these aforementioned variables; there was no association demonstrated between AC and recurrence in this model either (adjusted HR 0.93, 95%CI 0.63–1.38; *P* = 0.733).

Factors which were significantly associated with recurrence after adjusting for the competing risk of non-RC death included the need for en bloc excision of an adjacent structure (HR 1.95, 95%CI 1.24–3.06; *P* = 0.004); an operating time ≥ 4 h (HR 1.41, 95%CI 1.02–1.94; *P* = 0.038); patients with a T4 tumour (HR 1.81, 95%CI 1.17–2.78; *P* = 0.008); patients with a lymph node ratio ≥ 0.2 (HR 2.2, 95%CI 1.64–3.07; *P* < 0.001); poor tumour differentiation (HR 1.93, 95% 1.40–2.67; *P* < 0.001); and those with lymphovascular invasion (HR 1.84, 95%CI 1.35–2.52; *P* < 0.001).

### Rectal-cancer-specific death

Death due to RC occurred in 119 patients (35.2%) and death due to other causes in 59 17.5%), whilst 160 patients (47.3%) had censored survival times (Table 4). For the 208 patients who had chemotherapy, these numbers were 65 (31.3%), 18 (8.7%) and 125 (60.1%), respectively, and for the 130 who did not, 54 (41.5%), 41 (31.5%) and 35 (26.9%).

**Table 4:** Association between adjuvant chemotherapy (AC) and death due to rectal cancer and between other variables independently associated with administration of AC and death due to rectal cancer, after adjustment for the competing risk of death due to causes other than rectal cancer

	Category^a^	Censored *n* = 160	Died of non-RC *n* = 59	Died of RC *n* = 119	Bivariate hazard ratio (95% CI)	Wald *P*	Multivariable hazard ratio (95% CI)	Wald *P*
Adjuvant	No	35	41	54	REF	0.074		0.230
chemotherapy	Yes	125	18	65	0.72 (0.50–1.03)		0.77 (0.49–1.18)	
Year of resection	1995–1999	18	17	44	0.75 (0.63–0.89)	*0.001*	0.77 (0.65–0.92)	*0.004*
	2000–2004	33	23	27				
	2005–2009	41	12	30				
	2010–2014	24	5	16				
	2015–2019	44	2	2				
Age	< 75 years	142	30	95	REF	0.787		0.268
	≥ 75 years	18	29	24	0.94 (0.60–1.48)		0.74 (0.43–1.26)	
Peripheral vascular disease	No	159	53	110	REF	0.077		0.073
	Yes	1	6	9	1.90 (0.93–3.85)		1.92 (0.94–3.91)	
Intra-abdominal or pelvic abscess/haematoma	No	145	54	112	REF	0.381		0.203
	Yes	15	5	7	0.71 (0.33–1.53)		0.61 (0.29–1.31)	

Italicized *P* values indicate *P* significance at ≤ 0.05

*RC* rectal cancer, *CI* confidence interval

^a^In each cell, first line is the reference category coded 0, second line is the category of interest coded 1

**Fig. 2:**
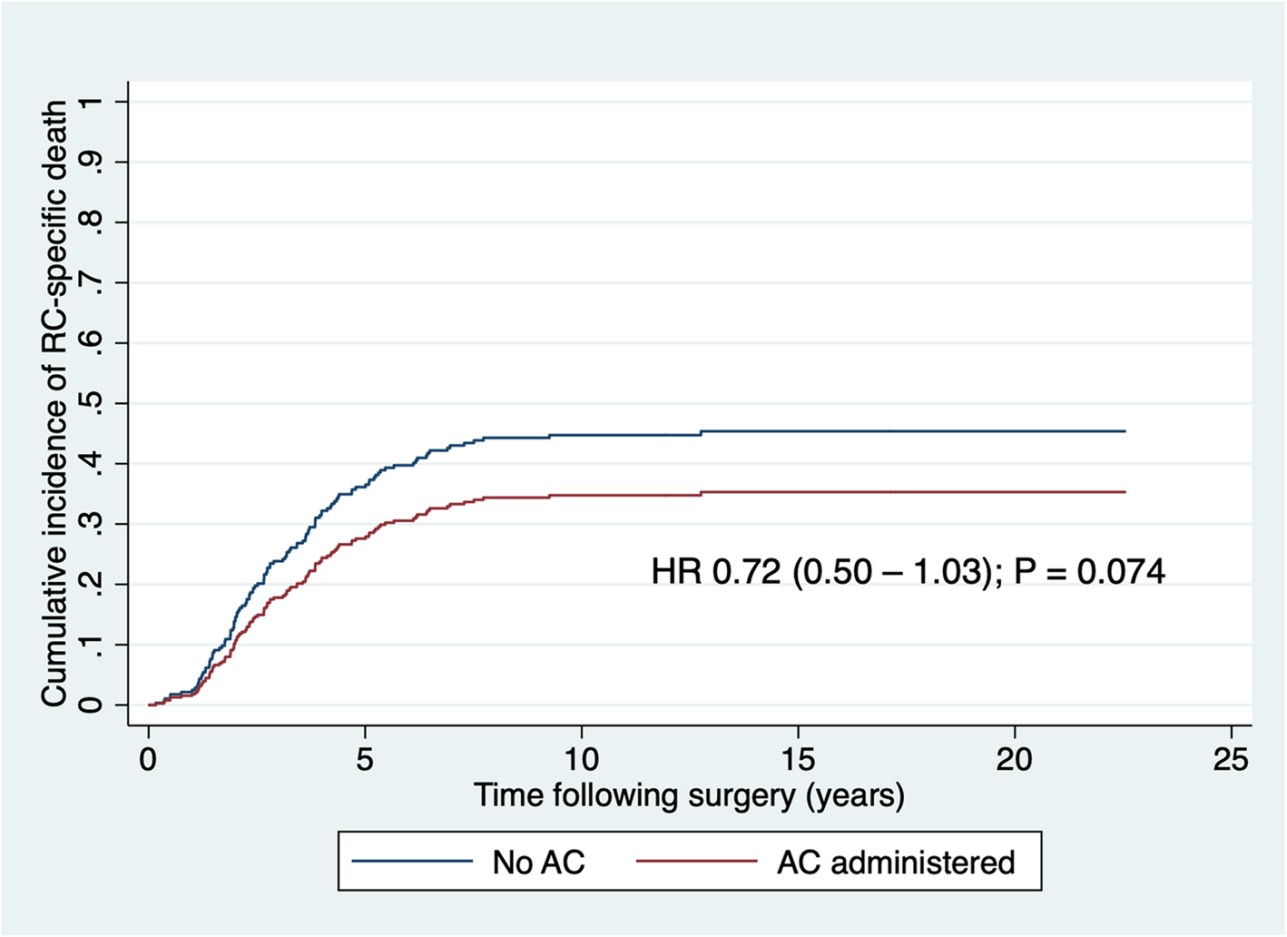
Cumulative incidence of rectal cancer-specific death after potentially curative resection by adjuvant chemotherapy

After adjustment for the competing risk of non-cancer death, death due to RC was not associated with AC, as shown in Table 4 and Fig. 2. To account for the possibility that one or more of the variables found to be independently associated with receiving chemotherapy confounded the association between chemotherapy and recurrence, a multivariate competing risks regression analysis was performed which incorporated specifically these variables and AC as predictors of RC-specific death. Notably, RC-specific death was associated with year of resection, with hazards of cancer-specific death decreasing through the study period (Table 4). After adjusting for this, there was still no association found between death due to RC and AC (adjusted HR 0.77, 95%CI 0.49–1.18; *P* = 0.230).

Factors which were significantly associated with RC-specific death after adjusting for the competing risk of non-RC death (apart from year of resection) included a need for en bloc excision of an adjacent tissue or organ (HR 1.80, 95%CI 1.06–3.04; *P* = 0.029); intraoperative blood loss ≥ 500 cc (HR 1.95, 95%CI 1.11–3.44; *P* = 0.021); T4 tumour (HR 2.16, 95%CI 1.36–3.43; *P* = 0.001); a lymph node ratio ≥ 0.2 (HR 2.56, 95%CI 1.77–3.68; *P* < 0.001); poor tumour differentiation (HR 2.52, 95%CI 1.72–3.68; *P* < 0.001); lymphovascular invasion (HR 1.68, 95%CI 1.17–2.40; *P* = 0.005); and where the tumour was located predominately on the anterior rectal wall (HR 1.47, 95%CI 1.02–2.10; *P* = 0.037).

### Overall survival

**Fig. 3:**
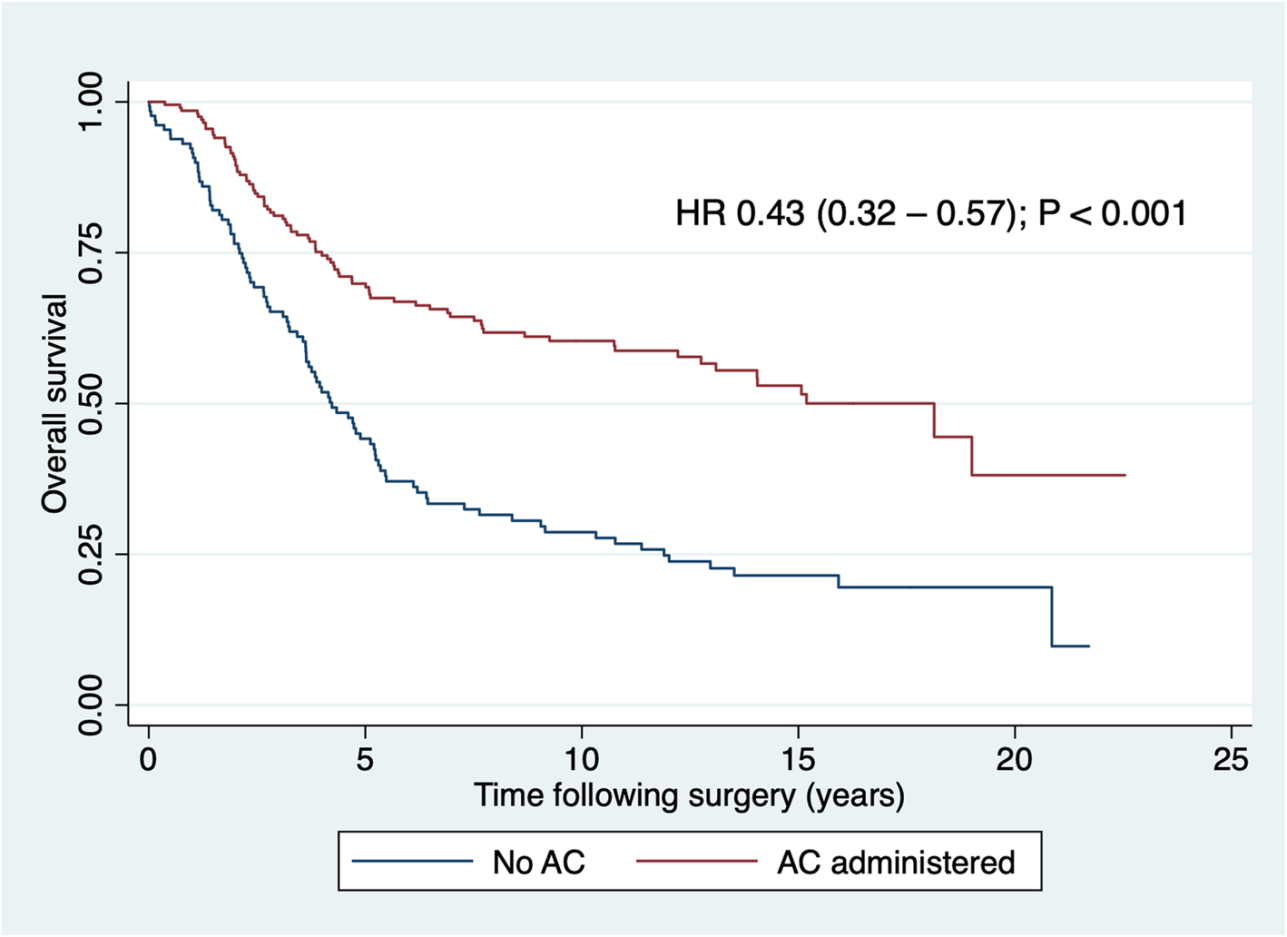
Overall survival after potentially curative resection of rectal cancer by adjuvant chemotherapy

OS was significantly longer in patients who received chemotherapy than in those who did not, as shown in Table 5 and Fig. 3. Also, OS improved as year of resection progressed, and shorter survivals were noted in patients aged 75 years and older and those with peripheral vascular disease. In a multivariable model, the associations with AC, year of resection, age and peripheral vascular disease all persisted (Table 5).

**Table 5:** Association between adjuvant chemotherapy and overall survival and between other variables independently associated with administration of AC and death due to any cause

	Category^a^	Censored *n* = 160	Died of any cause *n* = 178	Bivariate hazard ratio (95% CI)	Wald *P*	Multivariable hazard ratio (95% CI)	Wald *P*
Adjuvant chemotherapy	No	35	95	REF	*<0.001*		*0.009*
	Yes	125	83	0.43 (0.32–0.57)		0.61 (0.43–0.88)	
Year of resection	1995–1999	18	61	0.79 (0.69–0.91)	*0.001*	0.83 (0.72–0.96)	*0.013*
	2000–2004	33	50				
	2005–2009	41	42				
	2010–2014	24	21				
	2015–2019	44	4				
Age	< 75 years	142	125	REF	*<0.001*		*0.024*
	≥ 75 years	18	53	2.17 (1.57–3.00)		1.54 (1.06–2.26)	
Peripheral vascular disease	No	159	163	REF	*<0.001*		*0.003*
	Yes	1	15	3.39 (1.99–5.80)		2.30 (1.32–4.02)	
Intra-abdominal or pelvic abscess/haematoma	No	145	166	REF	0.833	–	–
	Yes	15	12	0.94 (0.52–1.69)			

Italicized *P* values indicate *P* significance at ≤ 0.05

*RC* rectal cancer, *CI* confidence interval

^a^In each cell, first line is the reference category coded 0, second line is the category of interest coded 1

### Disease-free survival

**Fig. 4:**
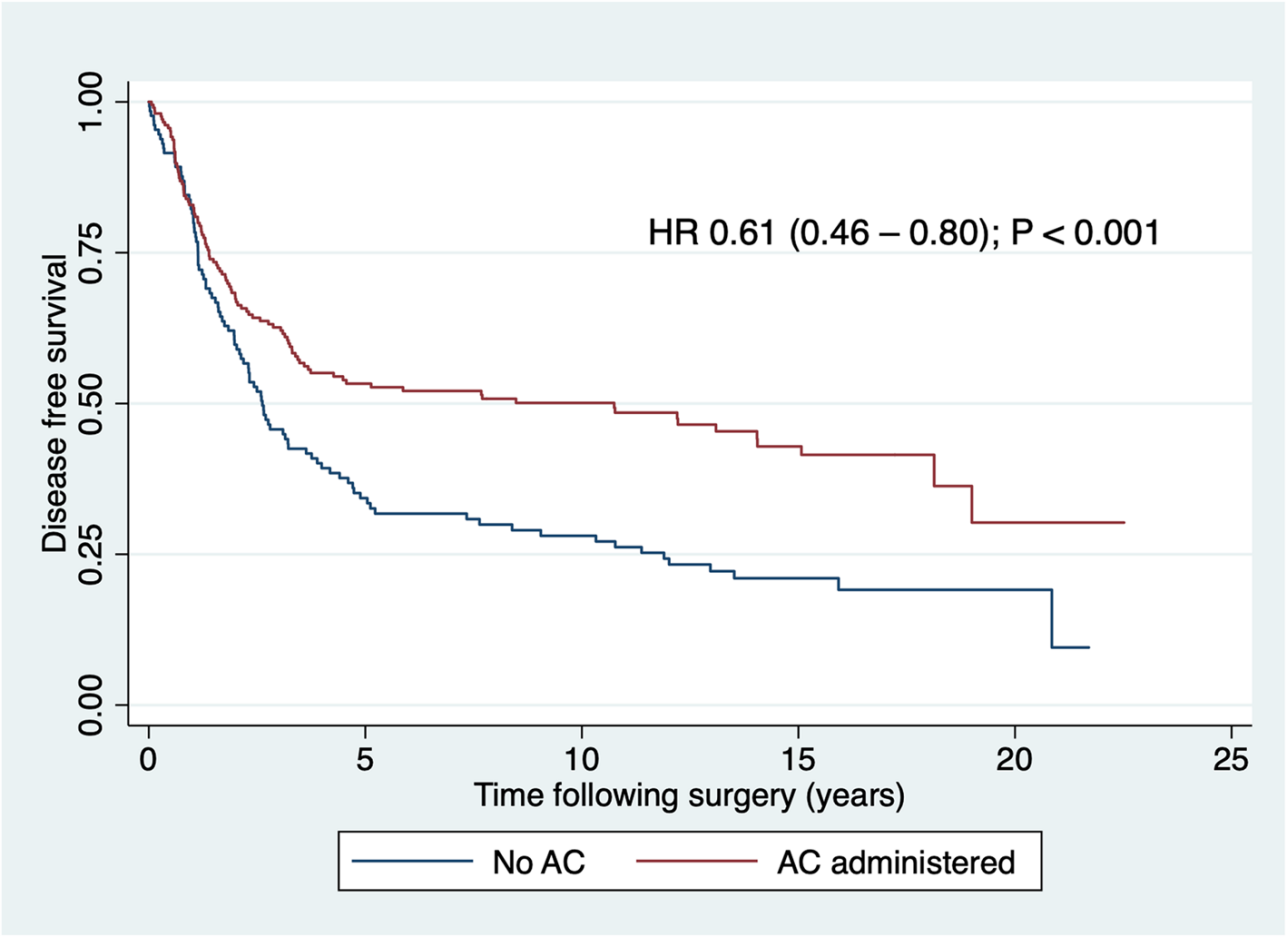
Disease-free survival after potentially curative resection of rectal cancer by adjuvant chemotherapy

DFS was significantly longer in patients who received AC than in other patients, as shown in Table 6 and Fig. 4, whereas patients aged 75 and older and those with peripheral vascular experienced shorter DFS than their counterparts. In a multivariable model, evidence for an association between DFS and AC attenuated whilst still remaining statistically significant, whilst associations with age and peripheral vascular disease disappeared (Table 6).

**Table 6:** Association between adjuvant chemotherapy and disease-free survival and between other variables independently associated with administration of AC and disease-free survival

	Category^a^	Censored *n* = 131	Died of any cause or recurred *n* = 207	Bivariate hazard ratio (95% CI)	Wald *P*	Multivariable hazard ratio (95% CI)	Wald *P*
Adjuvant chemotherapy	No	30	100	REF	*<0.001*		*0.048*
	Yes	101	107	0.61 (0.46–0.80)		0.72 (0.52–0.99)	
Year of resection	1995–1999	17	62	0.93 (0.83–1.04)	0.211	–	–
	2000–2004	29	54				
	2005–2009	34	49				
	2010–2014	20	25				
	2015–2019	31	17				
Age	< 75 years	117	150	REF	*0.001*		0.127
	≥ 75 years	14	57	1.69 (1.25–2.30)		1.32 (0.92–1.90)	
Peripheral vascular disease	No	130	192	REF	*0.004*		0.081
	Yes	1	15	2.17 (1.28–3.69)		1.63 (0.94–2.84)	
Intra-abdominal or pelvic abscess/haematoma	No	119	192	REF	0.824	–	–
	Yes	12	15	0.94 (0.56–1.59)			

Italicized *P* values indicate *P* significance at ≤ 0.05

*RC* rectal cancer, *CI* confidence interval

^a^In each cell, first line is the reference category coded 0, second line is the category of interest coded 1

## Discussion

This observational study of prospectively recorded data from 25 years of routine hospital practice, analysed by competing risks regression, found that AC did not result in significantly diminished recurrence nor reduced RC-specific death among the 208 consecutive stage III patients who received chemotherapy. Fifty variables which potentially could have influenced the selection of patients for chemotherapy or influenced oncological outcomes were examined, yet none was found to have a confounding effect to explain the absence of any impact from chemotherapy on recurrence or cancer-specific death. These findings are not directly comparable with the existing literature because no published studies have used recurrence or rectal-cancer-specific death as primary endpoints and none have used competing risks methods of analysis [6–12]. Rather, such studies used simply OS or DFS as endpoints. In our patients, OS was significantly longer in those who received chemotherapy, even after adjustment for relevant confounders. DFS was initially longer in those who received chemotherapy, but this difference attenuated to borderline statistical significance after adjustment for confounders.

In early cancer trials studying the impact of AC [32], recurrence was assessed by naïve Kaplan–Meier censoring [18] which treats competing events (such as non-cancer death) as censored observations. This results in inaccurate recurrence estimates, which is avoided by the use of the competing risks method [18–20]. In the present study, the use of competing risks methods showed an absence of any association between AC and recurrence as the principal endpoint. After multiple regression adjustment for the possibility of a suppressor effect from any factor which was related to patient selection to receive chemotherapy, there was still no indication of a significant effect.

In this study, only OS showed a significant independent protective effect of chemotherapy, a finding which is consistent with a previous population-based longitudinal study [33]. This seems difficult to reconcile with our finding of no association between chemotherapy and recurrence. The explanation lies in appreciating that recurrence is a necessary condition for cancer-specific death in a patient following resection of a stage III cancer; if the patient has not developed a recurrence, they cannot die of RC. In OS, the ‘failure event’ is death from any cause, so if chemotherapy is not associated with recurrence, and as recurrence is a necessary condition for cancer death, then diminished OS cannot be a consequence of not having chemotherapy but must arise from some other cause. Similarly, for DFS (or recurrence-free survival) where the principal failure event is either cancer recurrence or death from any cause [34], if chemotherapy is not associated with recurrence, then diminished DFS cannot be a consequence of not having chemotherapy but rather must arise from some other cause. In contrast, the outcome in competing risks analysis of recurrence is a single event (recurrence) rather than a combination of events (recurrence or death due to any cause). Unsurprisingly, the use of OS or DFS as endpoints would yield misleading results when there is no association between chemotherapy and recurrence in stage III RC.

Clinical practice guidelines published by the American Society of Colon and Rectal Surgeons [35] and the Association of Coloproctology of Great Britain and Ireland [36] both advocate that AC should be considered for patients with stage III RC if systemic chemotherapy has not been given preoperatively. However, both guidelines recognise that their recommendations are based on extrapolation of data from adjuvant colon cancer therapy given the paucity of data specifically for RC, especially those managed contemporarily with TME surgery and neoadjuvant therapy. Such uncertainty has led to the current recommendation by the (Australian) National Health and Medical Research Council that the uncertain benefits of AC should be acknowledged, even in patients regarded as ‘high-risk’ [37]. Undoubtedly, it would be desirable that modern trials be repeated based on contemporary surgical and medical management, the results of which could ‘replace’ those of dated trials which remain controversial. However, because of the widespread acceptance of AC and for ethical reasons, it is unlikely that new randomised trials will be conducted. Instead, future investigations on this topic would have to utilise prospective observational designs as in this present study, coupled with analysis of oncological outcomes by competing risks techniques. Also, such studies have the advantage of being conducted in routine practice rather than in the rarefied environment of a randomised trial.

A limitation of this retrospective study was that no detailed record was kept for reason/s why individual patients did or did not receive chemotherapy, nor of the dose alterations, complications or interruption or cessation of treatment. It is also acknowledged that chemotherapy regimens have changed over time, and more recent addition of oxaliplatin as doublet therapy may have influenced results. However, as chemotherapy treatment used established agents and was administered according to conventional guidelines at the time, it is difficult to explain how its application in routine clinical practice should not show at least some tendency toward a protective effect if such an effect is true.

## Conclusions

This study of prospectively recorded data on consecutive patients having a resection for stage III RC in a routine hospital setting found no significant difference in the prevalence of recurrence or cancer-specific death between patients who had received AC and those who had not. The failure to find a protective effect from AC may be attributable to standardized, anatomically based surgery with contemporary preoperative staging and multidisciplinary management. It is recommended that the endpoints of both cohort studies and randomised trials addressing this topic should consider both recurrence and cancer-specific death and that contemporary competing risks methods should be used in future analyses.

## Data Availability

The datasets used and/or analysed during the current study are available from the corresponding author on reasonable request.
